# The Association Between Willingness of Frontline Care Providers’ to Adaptively Use Telehealth Technology and Virtual Service Performance in Provider-to-Provider Communication: Quantitative Study

**DOI:** 10.2196/15087

**Published:** 2019-08-29

**Authors:** Hyeyoung Hah, Deana Goldin, Sejin Ha

**Affiliations:** 1 Department of Information Systems and Business Analytics Florida International University Miami, FL United States; 2 Nicole Wertheim College of Nursing & Health Sciences Florida International University Miami, FL United States; 3 Retail, Hospitality, and Tourism Management College of Education, Health, and Human Sciences The University of Tennessee, Knoxville Knoxville, TN United States

**Keywords:** telehealth technology, adaptive technology use, frontline care providers, virtual care service, daily habit of technology use, PLS modeling, telehealth, mhealth, ehealth, digital health, adaptive technology, frontline care, virtual care

## Abstract

**Background:**

Telehealth technology can create a disruptive communication environment for frontline care providers who mediate virtual communication with specialists in electronic consultations. As providers are dealing with various technology features when communicating with specialists, their flexible attitude and behaviors to use various telehealth-related technology features can change the outcome of virtual care service.

**Objective:**

The objective of this study is to examine frontline care providers’ technology adaptation behaviors in the electronic consultation context. From the perspective of frontline care providers, we reapply and retest a theoretical model, reflecting a mechanism through which technology users’ personal characteristics and technology adaptation behavior enhance virtual service performance, which is an important performance enabler in this online meeting context. In provider-to-provider communication, particularly, we explore the association among providers’ information technology (IT)–related personal characteristics, adaptive telehealth technology use, and virtual service performance.

**Methods:**

An online survey was administered to collect individual providers’ personal traits, IT adaptation, and perception on virtual service performance. Partial least squares-structural equation modeling was used to estimate our predictive model of personal traits—IT adaptation, such as exploitative use (use the telehealth technology in a standard way), and exploratory use (use the telehealth technology as innovative way)—and virtual service performance.

**Results:**

We collected 147 responses from graduate nursing students who were training to be nurse practitioners in their master’s program, resulting in 121 valid responses from the cross-section online survey. Our theoretical model explained 60.0% of the variance in exploitative use of telehealth technology, 44% of the variance in exploratory use of telehealth technology, and 66% of the variance in virtual service performance. We found that exploitative IT use is an important driver to increase virtual service performance (β=0.762, *P*<.001), and personal characteristics such as habit are positively associated with both exploitative (β=0.293, *P*=.008) and exploratory use behaviors (β=0.414, *P*=.006), while computer self-efficacy is positively associated with exploitative use of telehealth technology (β=0.311, *P*=.047).

**Conclusions:**

This study discusses the unique role of frontline care providers in a virtual care service context and highlights the importance of their telehealth adaptation behavior in provider-to-provider communication. We showed that providers perceive that telehealth technologies should function as intended, otherwise it may create frustration or avoidance of the telehealth technology. Moreover, providers’ habitual use of various technologies in daily lives also motivates them to adaptively use telehealth technology for improving virtual care service. Understanding providers’ technology habit and adaptation can inform health care policy and further provide a better view of the design of telehealth technology for online communication.

## Introduction

### Background

As telehealth technologies enable virtual and timely communication among care providers, frontline care providers particularly face challenges in enhancing service performance while using such technologies. In the primary care setting, care provider groups such as doctors, nurse practitioners, and nurses have been the first point of contact for people who seek health care services [1] within close proximity of patients in the location [2]. As the use of telehealth technology in electronic consultation (e-consultation) has expanded care providers’ role to managing some specialty care work [3,4] beyond locational boundaries [5], it has become visible how they broker specialty visits between primary and specialty care by using telehealth technology [6]. Telehealth technology is thus supposed to enhance frontline care providers’ virtual communication electronically. For example, care providers speak to patients via scheduled or on-demand/urgent visits; in addition, they communicate with specialists to ask questions and help patients avoid further face-to-face consultation with specialists [7]. Our main focus in this paper is on the latter case, often termed telespecialty consultation or e-consultation [8], which is the interaction between frontline care providers and specialists. In this environment, a patient typically does not see a specialist and relies solely on care providers' intervention to gain access to specialists, and thus, care providers’ need to manage each patient’s case in a timely manner while communicating with specialists. Thus, care providers are challenged to act as care moderators of the relationships between patients and specialists to manage expanded care responsibilities and improve service performance with the use of telehealth technology.

Such care providers’ moderating role requires them to adequately select and use telehealth technologies for successful virtual care outcome. Telehealth technology does not refer to a single technology artifact, but to a number of electronic information and communication technologies (ICTs) to facilitate long-distance clinical care, patient and professional health-related education, and public health administration [9]. Accordingly, prior literature has noted that care providers have increasingly used multiple technologies to manage not only the new form of health care, but also virtual communication simultaneously [10-12]. However, such use of multiple technologies for virtual services has led to mixed results. Care providers perceived the use of a single telehealth technology to be beneficial to the timely management of referrals to specialists, but at the same time, they felt burdened by the additional workload that had shifted from specialists [13]. In such processes, care providers’ increased use of other relevant technologies may create frustration and avoid adoption of new technology when certain technological features with which they are familiar do not perform as intended [14] or supplement the role of care providers [5]. More specifically, in the e-consultation context, care providers can feel constrained in sending messages to specialists if this familiar use of messaging technology is not integrated with other services or health systems [15]. Thus, it is fair to say that care providers’ prior use of various technologies and features and their expectation of telehealth technology influence their telehealth use behavior.

However, little attention has been devoted to understanding the telehealth-driven provider-to-provider communication in which individual providers’ technology use behavior as care moderators can influence virtual service performance. Several prior studies have mainly focused on the antecedents of virtual service performance [16], and yet, the mechanisms that influence care moderators’ perception on the use of telehealth technology and service performance in the process of e-consultation are unknown. Given that telehealth technology shares similarities with other health technologies on various devices such as smartphones, tablets, and desktop/laptop computers [17], care providers’ pre-existing experience and self-confidence in dealing with similar or new features from other technologies may not only affect their attitude about using telehealth technology [18], but also the way in which they use the telehealth technology for virtual communication [19]. In other words, a care provider may select and use a set of related telehealth technologies to manage online communication with specialists, which may allow them to enjoy familiar system features or may alter the intended capabilities of the telehealth technology artifacts (referred to as “adaptive use of IT” [20]).

Two aspects distinguish this study from prior studies. First, this study explicitly focuses on care providers’ postadoption behavior using a set of telehealth technologies. Thus far, extensive research has been performed at the intersection of human computer interaction (HCI), health informatics, design science, and information technology (IT) adoption strands, with the main focus on the cognitive/psychological aspect of technology use [21,22] and interactions with technology artifacts [23,24]. For example, prior research noted mechanisms through which individual care providers’ intrinsic and extrinsic motivation, gratification, and use environment influence adoption intention [25]. Additionally, exploration of how humans interact with social, organizational, and contextual environments has developed theoretical foundations to capture users’ technology adoption intention across nonhealth domains such as business, marketing, education, engineering, and agriculture (eg, [26]). This paper, however, examines an unexplored area of technology use—telehealth technologies and users’ level of flexibility—to mix, match, and use them for the success of telehealth care communication. This adds value to telehealth management and relevant research, given that the current technology market for telehealth is led by care providers, tech firms, and payers, and telehealth technologies may not share common features or include all necessary features for various care regimen [27]. For example, remote patient monitoring, as one of the important aspects of telehealth service, needs multiple technologies such as videoconferencing software, peripheral devices, telemedicine carts (filed kits) for the patient site, and remote patient monitoring kits [28]. In addition, care providers use audio and video technologies for live patient care [10]. In such a telehealth care environment, care providers play crucial roles in not only mediating as the first virtual contact for patients but also cocreating care plans with specialists. Their performance can thus affect the outcome of telehealth care services, which, in turn, influence telehealth technology adoption and use of other stakeholders in rural or medically underserved areas [29]. As a consequence, comprehending users’ (ie, care providers’) experiences with related technologies is essential to understand their telehealth technology behavior.

**Figure 1:**
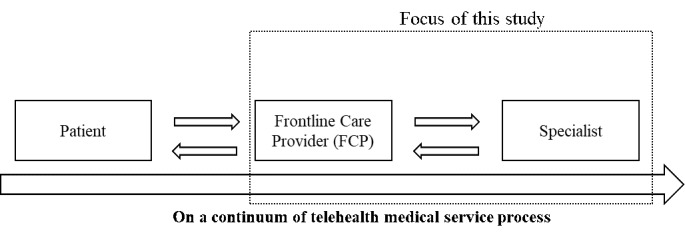
Focus of this study.

Second, this paper calls for an explicit focus on virtual communication between care providers and specialists. As telehealth service consists of multilateral communications among care stakeholders, primary care providers should moderate the encounters between those parties in case of virtual specialty care needed and enhance timely and quality of telehealth care. At the time of innovation, primary care providers are known to adapt their daily routines of care management to technological innovation, which leads to decreased productivity [30]. However, little is known about care providers’ ability to adapt telehealth technology for virtual communication and team-based care services [31]. From the perspective of frontline care providers who manage care processes in the location and connect to specialists remotely, the manner in which they evaluate the use of telehealth technologies and perform in this new care format can determine the success of telehealth care services in the long run. Figure 1 summarizes the unique role of frontline care providers in a telehealth care process and is the focus of this study. In this study, we limit our focus on variation in the provider-to-provider communication while making patient-side inputs fixed to better isolate care providers’ technology adaptation behavior.

Taken together, our aims are to examine whether care providers’ adaptive technology use behavior improves virtual service performance in e-consultation and to explore whether individual characteristics in relation to technology (personal innovativeness, computer self-efficacy, and habit) influence their adaptive use behavior. To this end, we examined two adaptive use behaviors: exploitative and exploratory use of telehealth technology. Exploitative technology use refers to the use of telehealth technology under the existing norms, while exploratory technology use involves the use of IT in a novel or unprecedented way. We hypothesized that these two adaptive IT behaviors enhance task performance on the part of care providers in the process of relaying patient information and managing specialists’ diagnoses and that personal traits may affect such adaptive IT use behaviors.

### Theoretical Background

#### Adaptive Use of Telehealth Technology

In this paper, we define telehealth technology as electronic ICTs that support both care management and various modalities of virtual care meetings. To explore care providers’ adaptive use of telehealth technology, we build on the Adaptive Structuration Theory (AST) at the level of individual users [20]. In Information Systems (IS) literature, AST explains constituents’ adaptive responses to technological changes and decision outcomes in an organization. The theory describes the mechanisms through which constituents make sense of organizational, technology-driven changes by selecting, adapting, and altering existing “social structures,” all of which lead either to group decision outcomes or create new structures within the organizational context [32-35]. Recently, Schmitz et al [20] extended these adaptation behaviors to the level of individual users within organizations by proposing individual-level social interactions with the focal technology and tasks. This theory states that social interaction processes can occur through two structuration episodes, including technology adaptation and task adaptation, where adaptation can be in two modes: exploitative and exploratory adaptation. Exploitative adaptation reflects the use of technology in line with existing norms and interpretations (expected use), while exploratory adaptation indicates technology use based on nonstandard interpretations (unexpected use). The dynamic effects of adaptive behaviors on individuals’ performance have been demonstrated in various research settings [20] such as job performance and satisfaction in nonhealth domains [36].

In health care, care providers’ adaptive use of telehealth technologies can play an important role in provider-to-provider communication. Weigel at al [37] defined individuals’ adaptive use of health information technology (HIT) as “temporary or permanent modifications that a user makes to his or her behaviors or norms due to the limitations of the HIT.” Thus, if technologies do not fully support e-consultation, this condition can elicit users’ adaptation behaviors [15]. In theory, the adaptive use of technology takes place when users have experienced various technologies and are expected to use existing or new technologies adaptively. In this case, the possible responses are either that they use a system feature as it is or modify some features to produce better outcomes. In the telehealth context, the adaptive use of telehealth technology is also expected, as health stakeholders increasingly use multiple technologies to communicate virtually with one another for care management beyond office visits. For example, care providers use personal messaging apps (eg, Whatsapp) and social network sites [11] to moderate communications between patients and care providers in addition to the designated telehealth technologies [7,38]. Hence, care providers’ adequate selection and use of other communication technologies may change the outcomes of telehealth services. In IS literature, such a moderating role of care providers is analogous to that of online meeting facilitators, whose behaviors not only affect the meeting outcomes, but also the other meeting attendees’ behaviors [39]. More specifically, there are two important factors that affect online meeting outcomes: one is facilitators’ personal characteristics such as their level of experience and facilitation [40], and the other is their technology-based skills [41-43]. In this paper, we focus on care providers’ technology-based skills, ie, technology adaptation behaviors and technology-driven personal traits as key determinants to influence virtual service performance.

#### Virtual Service Performance

This study considers virtual care performance to be one of the virtual meeting outcomes that captures care providers’ expected outcomes in response to adequate use of telehealth-related technologies in the e-consultation context. In general, the success of IT use or performance has been considered at multiple levels, such as at the individual, group, and organizational levels [44,45], and individual performance has been widely studied as a key dependent variable to measure individual users’ postadoptive IT use behavior [46-48]. Applied to our research context, care performance from primary care services has been measured by patients’ satisfaction [49], care providers’ evaluation of care coordination [50], or organizational performance [51]. In fact, telehealth technologies need to be able to support various communication modalities via video-conferencing, texting, and a combination of both during care provision [52]. Hence, it is critical that care providers apply effective virtual communication skills when managing electronic patient data [53] and use various technological features, all of which depend upon their proper choice and actual use of the technologies. Thus, we suggest that care providers’ virtual service performance be determined by the adaptive use of telehealth technologies, and their performance can be improved with respect to effectiveness of care, care management, quality of care tasks, decreased error rates in communication, and sharing information [54].

#### Personal Innovativeness in Information Technology, Computer Self-Efficacy, and Habit

Care providers’ responses to these challenges can be reinforced or redirected by their personal characteristics. Prior studies on information technology use have documented the importance of personal characteristics that help users experiment with and control new technology based on their beliefs and experience. When IT users communicate with others online, in general, and when they act as meeting facilitators in a virtual meeting, in particular, the user who presides over the meeting becomes more important, because individual characteristics such as technology-based skills, capabilities, and level of experience [55] affect the success of online meetings and meeting members’ use of technologies [11,38]. Given that care providers need to facilitate virtual communication with specialists, these users’ characteristics and beliefs about the use of telehealth and relevant technologies simultaneously help to predict the online communication outcome, which is virtual service performance in this study. Thus, care providers’ willingness and capability to use multiple telehealth-related technologies (including familiar and new features) and whether care providers possess characteristics that allow them to introduce more innovative ways of technology use can influence virtual service performance [56].

The literature on postadoptive technology, behavior has acknowledged that users’ personal characteristics are important antecedents that explain their postadoption behavior [57,58]. In this model, we identified personal innovativeness in IT use, computer self-efficacy, and habit as determinants to explain care providers’ adaptive telehealth technology use behaviors. First, personal innovativeness in IT use is defined as “the willingness of an individual to try out any new information technology” [57]. This concept has been widely used in IT use studies to capture individuals’ intention to adopt technology, both generally [59] and in health care domains [60-62]. In the adaptive use context, Chow et al [63] found that personal innovativeness positively influenced adaptive IT use behavior. The higher innovativeness a user has, the more likely that he or she is to try new features and mix and match system features that are relevant to tasks (eg, by replacing some existing features with new ones, combining features, or inventing new ways to use certain features for tasks for which they were not intended). Second, computer self-efficacy—referring to a user’s belief about his or her capability to control telehealth technologies—is also likely to influence IT users’ motivation and outcomes [64,65]. Users’ self-judgments about technology efficacy influence their beliefs about a focal technology’s ease of use [65]. In postadoption IT use, individual users’ beliefs about their ability to use new technology are associated with the technology’s deep structural use [66,67]. Lastly, habit concerns the notion that the “habitualization of action occurs more or less automatically via a subconscious response to a work situation” [68]. Thus, people may be willing to adopt a new workplace technology when they understand other technologies in their lives. As automatic reactions to certain tasks that are attributable to prior learning, habits have been identified as predictors of technology adoption or moderators that interact with other factors in postadoption IT use [69-71]. Moreover, habits have been associated with continued use of IT [72,73] and its adaptive use [74]. Schmitz [20] used experience of technology as a personality trait, whereas we used habit instead. This is because habit captures an automatic reaction to certain tasks due to prior learning from technology, while experience reflects users’ exposure to a focal technology in the passage of time [68]. As we focus on users’ prior learning from the use of various technologies, habit is more applicable to our telehealth care context. Taken together, individual characteristics act as key antecedents that predict care providers’ adaptive use behaviors.

### Research Model and Hypothesis Development

#### Exploitative Use of Telehealth Technologies and Virtual Service Performance

According to Schmitz [20], exploitative use of focal technology occurs “when a user modifies technology features to facilitate usage of the technology consistent with how s/he perceives is intended or standard for the technology.” Thus, exploitative IT use reflects the routine use of IT under existing norms and expectations [36]. Exploitative use of technology occurs in various settings. For example, users employ IT in repetitive tasks to improve efficiency [75], complete tasks [76], and maximize task performance [77]. In a provider-to-provider context, care providers use personal messaging apps (eg, Whatsapp) to expedite communication with specialists after submitting e-consultation requests on telehealth technology platforms [11]. In this case, combined use of the telehealth technology and personal messaging applications that accomplish repetitive tasks can facilitate instantaneous communication with specialists, all of which influence virtual care performance. Thus, we hypothesize the following:

Hypothesis 1: Exploitative use of telehealth positively affects virtual care performance.

#### Exploratory Use of Telehealth Technologies and Virtual Service Performance

Exploratory technology use takes place “when a user develops new technology features to facilitate usage of the technology that s/he perceives is unusual or non-standard for the technology” [20]. Nontraditional IT use allows users to identify certain new capabilities of IT, such as exploring new skills and experimentation [76], and to make nonstandard interpretations of the focal phenomenon, leading to divergent consequences [20]. Accordingly, exploratory use of telehealth technology for e-consultation indicates an innovative use of telehealth technology that fosters deviation from existing tasks and the search for alternatives [77]. For example, to achieve timely communication with specialists, care providers need to be capable of managing images and reports and interacting with their electronic health records, which are typically accessed through their mobile phones [78]. Finding ways to quickly process multimedia images or connect hospital systems via interface applications installed on care providers’ devices might be examples of the exploratory use of telehealth technologies. Therefore, we made the following hypothesis:

Hypothesis 2: Exploratory use of telehealth positively affects virtual care performance.

#### Individual Characteristics as Antecedents of the Adaptive Use of Telehealth Technologies

**Figure 2:**
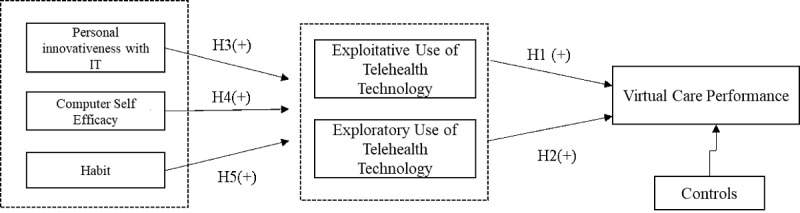
Research model. H: hypothesis; IT: information technology.

This paper proposes that personal traits are formulated via cumulative exposure to various technologies across multiple life domains (such as the workplace and at home) and hypothesizes that such traits can expand care providers’ capabilities to use both existing and new features in telehealth-related technologies. Individual care providers’ existing beliefs about self-innovativeness, self-judgment about telehealth technology use, and accumulated habits from using daily technologies across multiple life domains can influence the way in which they adaptively use new features of telehealth technologies in both expected and reconfigured ways, particularly for communication with providers (Figure 2). Therefore, we made the following hypotheses:

Hypothesis 3: Care providers’ personal innovativeness with IT positively affects their adaptive (H3a: exploitative; H3b: exploratory) use of telehealth technologies.

Hypothesis 4: Care providers’ computer self-efficacy positively affects their adaptive (H4a: exploitative; H4b: exploratory) use of telehealth technologies.

Hypothesis 5: Care providers’ habits with regard to technology positively affect their adaptive (H5a: exploitative; H5b: exploratory) use of telehealth technologies.

## Methods

### Recruitment

We recruited graduate nursing students at a university in the Southeastern United States who were studying to become family nurse practitioners and had experienced various types of technologies across different contexts. In telehealth care contexts, in particular, the demand of nurse practitioners is growing because they can collaborate with other specialists to comanage patient care cases for rural or disadvantaged population [42]. With incorporation of such trends and our research focus on nurse practitioners’ role as frontline care providers in e-consultations with specialists, we had to use purposive sampling for those who could provide unique information about telehealth care services that could not be obtained elsewhere [79]. The participants had been trained in the use of telehealth technologies in their education program and their communication with other care members in the simulation rooms, in which the nursing faculty had recorded and evaluated their electronic communication. In summary, our student sample could be legitimately used to capture their adaptive use of telehealth technologies. This nonprobability sampling met our goals to identify a representative sample of nurse practitioners who would be at the front line of telehealth care services as care providers (see [78] for the taxonomies of purposive sampling). To test our hypotheses, a Web-based survey was administered in the spring and fall semesters in 2018. In the online survey, each participant read a case scenario and responded to the questionnaire in terms of their perception on prior experience, adaptive use of telehealth technology, and care service performance (Multimedia Appendix 1).

### Statistical Analysis

#### Instrument Development

The measurement items were adopted from well-established IS literature and adapted to the telehealth context (Multimedia Appendix 2). To measure the dependent variable, we adapted Goodhue’s [47] five-item scale of task performance. This measure has been used to explain IT postadoption performance in various contexts, including health care [58]. Two independent variables—exploitative and exploratory use—were measured using items from a previous study [20], in which each item was assessed with four indicators. With regard to the input variables (antecedents to postadoptive IT use), personal innovativeness in IT was measured using three items adapted from a prior study [57]. Computer self-efficacy (a user’s perception of his or her competency in using computers) was measured using four items developed by Compeau and Higgins [64]. Habit reflects care providers’ automatic reactions to a new technology based on prior experience and was captured using four items [68]. Finally, we collected demographic variables (age, income, education, gender, occupation status, and experience in using health apps) and mobile technology usage experience and treated them as control variables in the data analysis. All research variables were reflective constructs and measured on a seven-point Likert scale that ranged from 1 (“strongly disagree”) to 7 (“strongly agree”). To assess face validity, the researchers contextualized the survey scale to make it pertinent to the telehealth care situation. A pretest was performed to ensure content validity. The researchers and two nurse practitioners evaluated and refined each survey item. A total of 37 initial responses were used to revise and finalize the questionnaire for the final survey.

#### Analysis

This study used composite-based partial least squares-structural equation modeling (PLS-SEM) in SmartPLS (Version 3.0. Boenningstedt, Germany: SmartPLS GmbH) to assess the measurement and structural models [80]. PLS-SEM is a causal model that has been frequently used in IS literature [81,82] and is used to maximize the variance that the dependent latent constructs explain [83]. We applied a bias-corrected and accelerated bootstrapping procedure with replacement using 5000 subsamples. Our hypotheses were tested using a one-tailed *t* test for unidirectional hypotheses.

## Results

### User Characteristics and Descriptive Statistics

A cross-sectional online survey was sent to 210 students who were enrolled in the nurse practitioner education program. A total of 146 participants responded to the survey, yielding a response rate of 69.52%; of these, 121 valid responses were used for data analysis. Twenty-five responses were dropped because they only reported demographic information in the surveys. Table 1 summarizes the respondent profiles. The majority were females (72.7%) and employed (full**-**time workers=54.5%; part**-**time workers=35.5%). All were well educated, with an undergraduate or higher degree, and nearly half were of white race (47.5%). Table 1 summarizes the characteristics of the survey participants.

In addition, an adequate sample size is necessary to estimate the PLS path model, which is guided by the 10-times rule and power analysis. On one hand, the 10 times rule dictates the use of “10 times the largest number of formative indicators used to measure a single construct, or 10 times the largest number of structural paths directed at a particular construct in the structural model” [83]. On the other hand, power analysis provides a threshold for the statistical power necessary to detect an effect based on the maximum number of independent variables in the measurement and structural models. In our case, we had five independent variables, three maximum arrows pointing to a latent construct, and thus needed at least 45 observations to achieve a statistical power of 80% and *R*^2^ values of at least 0.25 (with a 5% probability of error) [83]. Thus, our sample size (N=121) was deemed adequate to test the research model.

**Table 1:** Participant characteristics (N=121).

Demographic variables		Values, n (%)
**Gender**		
	Male	33 (27.3)
	Female	88 (72.7)
**Age (years)**		
	18-25	12 (9.9)
	26-40	85 (70.2)
	41-55	20 (16.5)
	56-65	4 (3.3)
**Income status (US $)**		
	25,000-49,999	24 (19.8)
	50,000-74,999	48 (39.7)
	75,000-99,999	16 (13.2)
	≥100,000	13 (10.7)
	Prefer not to answer	20 (16.5)
**Education**		
	Bachelor’s degree	72 (59.5)
	Master’s degree	41 (33.9)
	PhD	2 (1.7)
	Others	6 (5.0)
**Occupation^a^**		
	Working full time	66 (54.5)
	Working part time	43 (35.5)
	Unemployed	8 (6.6)
	Unable to work	1 (0.8)
	Other	3 (2.5)
**Race^b^**		
	African American	21 (17.5)
	Asian	18 (15.0)
	Native Hawaiian or Pacific Islander	1 (0.8)
	White	57 (47.5)
	Other	19 (15.8)
	Prefer not to answer	4 (3.3)

^a^All demographic questions were optional. Four respondents reported their occupation status as either “unable to work” or “other.” For clarity, we removed these responses and reran partial least squares analysis, producing the identical results.

^b^N=120.

### Nonresponse Bias and Common Method Bias

As our survey was self-reported, we evaluated two possible biases carefully: nonresponse bias and common method bias. Nonresponse bias derives from the differences between participants and nonparticipants in the survey [84,85]. This bias can be assessed by comparing our sample’s characteristics with those in the population and by comparing early and late respondents. We compared the early respondents (74.82%) and late respondents (25.18%) on each of the demographic characteristics (age, gender, education, income, and occupational status) and health application experience using a *t* test. There were no significant differences between the early and late respondents in our sample.

Common method bias potentially threatened the veracity of our results, as data for the independent variables and dependent variable were collected in the same survey. Following a previous study [86], we designed the survey instrument’s contents and order carefully. Furthermore, we performed the Harman single factor analysis to assess the bias. The results showed that one factor explained 36.90% of the variance, confirming that no single factor accounted for the majority of covariance. Thus, nonresponse bias and common method bias did not threaten this study’s findings.

### Measurement Model

To determine each construct’s internal reliability, we first examined the item loadings and composite reliabilities. Each item loaded above 0.75 on its respective construct and was significant at *P*<.05. Cronbach alpha was calculated to assess composite reliability and confirmed that all items’ values were above 0.7. Convergent validity was established if the average variance extracted (AVE) was above the threshold of 0.5, which suggests that the variance explained by indicators was greater than the unexplained variance. Discriminant validity was tested by assessing the Fornell-Larcker criterion and cross-loadings. To confirm the discriminant validity, the AVE of each construct should be greater than its squared correlations with other constructs [87]. As shown in Tables 2 and 3, internal reliability, convergent validity, and discriminant validity were all confirmed.

### Hypothesis Testing: Partial Least Squares Modeling

Following structural model assessment suggestions of de Guinea [69], we evaluated the proposed path model using SmartPLS 3.0. The structural model’s quality was assessed by checking multicollinearity (variance inflation factor), path coefficients, *R*^2^ (variance explained), *f*^2^ (effect size), and the Stone-Geisser *Q*^2^ (model’s predictive relevance). The variance inflation factor was checked and confirmed to be less than 5, indicating that multicollinearity was not a problem in the study. We report the path coefficients’ significance, *R*^2^ and *f*^2^ in the full model results. Effect size (*f*^2^) explains the changes when an exogenous construct of focus is included and when it is omitted from the model. As a rule of thumb, if *f*^2^ is 0.02, 0.15, and 0.35, the effects are considered to be small, medium, and large, respectively [88]. Lastly, to assess the model’s predictive power, the Stone-Geisser Q^2^ was used to indicate the sample’s predictive relevance. A *Q*^2^ value>0 demonstrates that the path model has predictive relevance to a reflective, endogenous latent variable. The *Q*^2^ values for three of the endogenous constructs were >0, indicating exploitative use (*Q*^2^=0.51), exploratory use (*Q*^2^=0.36), and virtual service performance (*Q*^2^=0.54). Thus, the model’s predictive relevance was confirmed.

**Figure 3:**
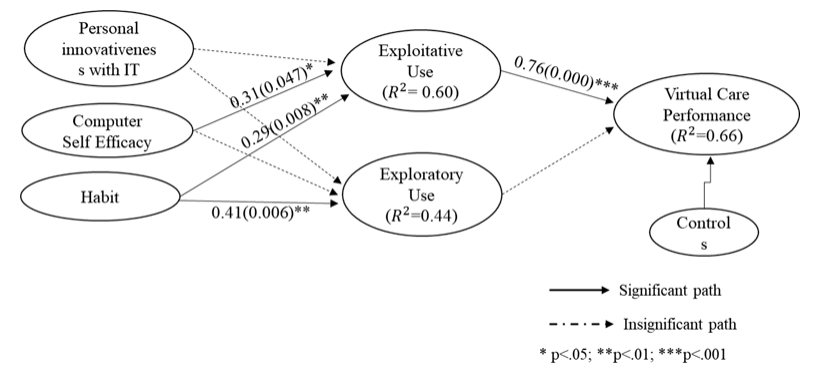
Structural evaluation of the telehealth adaptive use model. IT: information technology.

For the path coefficients’ significance and the variance explained, *R*^2^, our results demonstrated that exploitative technology use was positively associated with virtual service performance (β=0.76, *P*<.001), while exploratory use did not explain the variation in virtual service performance (β=0.036, *P*=.49). With regard to the effects of individual characteristics, computer self-efficacy was significantly associated with exploitative technology use (β=0.31, *P*=.05). Lastly, habit was associated with both exploitative (β=0.29, *P*=.04) and exploratory use (β=0.41, *P*=.006). Therefore, hypotheses 1, 4a, 5a, and 5b were supported but hypotheses 2, 3a, 3b, 4b, and 5b were not supported. Among the control variables, education (β=–0.12, *P*=.01) was shown to affect virtual service performance negatively, while income level (β=0.11, *P*=.008) was positively associated with virtual service performance, as shown in Table 4 and Figure 3.

**Table 2:** Internal and convergent validity.

Construct and items		Factor loading	Cronbach alpha	Average variance extracted	Mean (SD)
**HAB^a^**			0.96	0.89	5.38 (1.22)
	HAB1	0.95			
	HAB2	0.92			
	HAB3	0.94			
	HAB4	0.96			
**PIT^b^**			0.97	0.94	4.67 (0.86)
	PIT1	0.97			
	PIT2	0.96			
	PIT3	0.97			
**CSE^c^**			0.97	0.92	5.90 (0.96)
	CSE1	0.94			
	CSE2	0.96			
	CSE3	0.96			
	CSE4	0.96			
**EIU^d^**			0.98	0.94	5.92 (1.14)
	EIU1	0.98			
	EIU2	0.98			
	EIU3	0.99			
	EIU4	0.92			
**ERU^e^**			0.96	0.9	4.78 (1.52)
	ERU1	0.92			
	ERU2	0.94			
	ERU3	0.96			
	ERU4	0.96			
**PERF^f^**			0.97	0.9	5.63 (1.22)
	PERF1	0.95			
	PERF2	0.97			
	PERF3	0.97			
	PERF4	0.96			
	PERF5	0.89			

^a^HAB: habit.

^b^PIT: personal innovativeness with information technology.

^c^CSE: computer self-efficacy.

^d^EIU: exploitative use.

^e^ERU: exploratory use.

^f^PERF: care performance.

**Table 3:** Discriminant validity. Diagonals represent the value of the average variance extracted, and off-diagonal elements are the squared correlations among construct.

Constructs	1	2	3	4	5	6
1. Care performance	0.95	—^a^	—	—	—	—
2. Computer self-efficacy	0.59	0.96	—	—	—	—
3. Exploitive use	0.80	0.74	0.97	—	—	—
4. Explorative use	0.64	0.60	0.79	0.95	—	—
5. Habit	0.61	0.86	0.74	0.65	0.94	—
6. Personal innovativeness with information technology	0.59	0.86	0.73	0.62	0.87	0.97

^a^Not applicable.

**Table 4:** Complete results of the hypothesis testing.

Path		β^a^	SD	*t* test	*P* value	*f* ^2b^	Effect size	Hypothesis testing
**Virtual service performance**								
	H^c^1: Exploitive use	0.762	0.075	10.16	<.001	0.483	Large	Supported
	H2: Exploratory use	0.036	0.049	0.69	.49	0.001	—^d^	Not supported
**Exploitative use**								
	H3a: Personal innovativeness with IT^e^	0.201	0.134	1.531	0.13	0.019	—	Not supported
	H4a: Computer self-efficacy	0.311	0.155	1.991	0.047	0.05	Small	Supported
	H5a: Habit	0.293	0.112	2.648	0.008	0.042	Small	Supported
**Exploratory use**								
	H3b: Personal innovativeness with IT	0.168	0.155	1.095	0.27	0.01	—	Not supported
	H4b: Computer self-efficacy	0.102	0.149	0.672	0.5	0.004	—	Not supported
	H5b: Habit	0.414	0.15	2.781	0.006	0.06	Small	Supported
**Control variables**								
	Age	0.01	0.06	0.11	0.91	0	—	N/A^f^
	Education	–0.12	0.05	2.44	0.02	0.031	Small	N/A
	Gender	–0.09	0.05	1.67	0.1	0.009	—	N/A
	Income	0.11	0.04	2.65	0.01	0.02	Small	N/A

^a^Standard regression coefficient.

^b^Effect size.

^c^H: hypothesis.

^d^Not available.

^e^IT: information technology.

^f^N/A: not applicable.

## Discussion

### Principal Findings

The purpose of this study was to contextualize and test a research model that examined the determinants of the adaptive use of telehealth technologies in e-consultation. Using AST at the individual level, we examined the mechanisms by which exploitative and exploratory use of telehealth technologies and three personal traits (ie, personal innovativeness in IT, computer self-efficacy, and habit) influence virtual service performance.

Our results indicate that care providers are willing to use telehealth technologies exploitatively to communicate with specialists in e-consultations and perceive it as a virtual service performance enhancer. With regard to the hypotheses on adaptive use and virtual service performance (hypotheses 1 and 2), exploitative use of telehealth technologies was found to be a strong factor that explains care providers’ virtual service performance (β=0.762, *P*<.001), while exploratory use was not (β=0.036, *P*=.49). Moreover, with regard to hypotheses 3 through 5, we found that personal innovativeness in IT is insignificant for explaining adaptive use in our context (*P*=.13 for exploitative use and *P*=.27 for exploratory use, respectively); computer self-efficacy has a significant, positive effect on the exploitative use of telehealth technologies (β=0.311, *P*=.047); and habitual use of nonhealth technologies in daily life is associated with care providers’ willingness to engage in both exploitative (β=0.293, *P*=.008) and exploratory adaptive use of new telehealth technology (β=0.414, *P*=.006).

### Limitations

Despite the meaningful and practical findings in this study, our results should be interpreted with caution due to some limitations. First, we selected a purposive sample of graduate nursing students for our study. Although this is a legitimate sample, the ability to generalize the findings may be limited because our participants have experienced e-consultation in the education-focused, simulated contexts. Second, while our cross-sectional sample shows adequate responses to estimate our hypothesized path model using PLS-SEM, a larger sample with a panel structure would increase the statistical power of the findings and control for any unobserved confounding factor using panel data analysis. Particularly, time-series cross-sectional data on capturing various technology use among care providers would provide more in-depth understanding of virtual telehealth care services. Third, we did not include task adaptation behavior based on our research context’s characteristics. The education program requires individuals to adhere to the standard protocol of patient cases, such that any adaptive task behaviors are evaluated as a failure in the medical setting. Thus, we limited our focus to the participants’ adaptive use of telehealth technology only. It would be meaningful to investigate task and technology adaptation behaviors in the health care contexts in which the selection of tasks and technologies are flexible in future research. Fourth, this research considered care providers’ perception about their willingness to use telehealth technology either in a traditional way or in an innovative way and its downstream effect on perceived service performance. This is because we viewed telehealth technology as a set of related information and communication technologies and asked participants’ adaptation behavior in a general context. It would be worthwhile to revisit our research model to certain care contexts (eg, diabetes) and capture actual measures of virtual service performance. Lastly, future research can be further extended to explore other stakeholders’ attitude and behaviors about telehealth technology in e-consultation. For example, our research model can explain how adaptive use of telehealth technology by frontline care providers influences the level of patients’ satisfaction and health outcomes as well as those of specialists.

### Comparison with Prior Work

This study contributes to both the IS and health care literature on the postadoptive use of telehealth technology. First, this research contributes to the IS literature by exploring adaptive IT use behavior from an individual meeting facilitator’s perspective and attempting to identify a contextualized theory in e-consultation. Specifically, we considered e-consultations between care providers and specialists in a virtual meeting context and proposed that as virtual meeting facilitators, care providers’ willingness to use telehealth technologies is an important predictor of virtual service tasks’ success. Previous IS literature on online meeting technology use has documented that technology types, environmental factors, and user characteristics are key factors that predict outcomes from a multilevel perspective. From a group perspective, technical support for group users and the fit between tasks and technologies are important determinants of the success of online meeting technology use [89]. In terms of individuals’ behavior, extant studies have emphasized the important role that human facilitators’ characteristics play in predicting the outcomes of online meetings [43]. Given care providers’ unique position as those who relay clinical information and decisions between patients and specialists in health care and the availability of flexible options to select and use telehealth technologies for provider-to-provider communication, there is an urgent need to examine whether, and in what way, such meeting facilitators’ adaptive use of IT predicts the success of virtual service performance.

Our study is unique in that we focused explicitly on care providers’ virtual role in telehealth communication and their performance under a new definition of telehealth technology (ie, use of telehealth and telehealth-related technologies). Care providers’ role differs from that of general online facilitators in online meetings, in that they manage multilateral communication between patients and specialists and although there is a designated telehealth platform for their communications, sometimes, communication with the two different groups involves the use of additional technologies that complement or substitute the existing technology’s capabilities. Given that providers [58] and patients [90] were the main user groups of interest in previous studies for predicting telehealth technologies’ success, this study contributes to the health care literature by examining the adaptive structuration theory of individual from a care provider’s perspective and identifying salient constructs (the exploitative use of IT) in a research model to develop a contextualized theory. Second, the result that habit plays a significant role in adaptive IT use calls for more attention in IS research on how users’ habits accumulate from different life domains to affect their postadoptive use of multiple technologies [91]. Previous postadoption studies have established that habit reflects the extent to which people tend to perform behaviors automatically because of past learning [69,92-95]. However, the relationship between habit and the continued use of IT has been mostly tested in a single domain; for example, mobile phone habits predict mobile phone use [93] and mobile internet habits affect mobile internet technology use [95]. In a health care setting, however, our findings emphasize that cross-domain, habitual IT use influences care providers’ adaptive technology behaviors. Our interviews with two nurse practitioners in family medicine also reflected these positive effects of the habitual use of technology in nonwork domains. One nurse practitioner stated,

My use of personal non-health-related technology gives me hope that I am computer savvy and would be able to learn new computer technology that is used in patient settings.

Another said:

Because of technology, I am able to see who is waiting in patient rooms and who is still waiting in the waiting room. When the numbers are high, it makes […] clinician[s] want to work faster so that they are not too delayed.

As telehealth medical services include three different communication modalities (video-conferencing, texting, and a hybrid of the two), care providers’ existing technology habits can help them select and use these three forms of communication and manage communication with patients and other providers.

Moreover, we found a strong effect of exploitative use (expected use of telehealth technology) on virtual service performance by frontline care providers. These findings are in line with those of a previous study [20], such that two modes of IT adaptation behaviors (exploitative and exploratory use) are differentially salient across research contexts. In other words, exploitative and exploratory adaptation behaviors may not coincide under the same context because users have different coping strategies toward information technologies [96]. For example, explorative use of technology became salient in context of mobile technology in the BYOD (bring your own device) context [20]; in the contexts of enterprise resource planning and product lifecycle management system use, exploratory as well as exploitative adaptation were differentially significant, contingent upon input factors [36]. In our research context, the strong effect of exploitative use of telehealth technology may be due to the contextual characteristics of care process. Actually, health care is a controlled and highly concentrated environment such that care providers expect the technologies to function as expected by supplementing their clinical tasks [5]. Since this study explored technology-related traits as antecedents to technology adaptation behavior in provider-to-provider communication, it is much anticipated that such technology adaptation behavior can vary by different contexts and heterogenous technology users.

Lastly, education and income were shown to differentially influence virtual service performance. Prior studies have documented negative effects of demographic variables on technology use, as less educated participants may have less knowledge, whereas those who earn less income may have less opportunities to access advanced information technology [97,98]. In our research context, we can interpret that the current level of technology education from bachelor’s degree may not be on par with specifics of telehealth technology use for communication with specialists. In addition, our graduate nursing students with high income level may have been exposed to various technologies within and outside the education program or care settings. Thus, demographic characteristics of individual users need to be included when exploring technology adaptation behavior.

### Practical Implications

This study’s findings can be applied to inform health care practitioners and health app designers. Strategic IT management is necessary for care providers who serve as virtual meeting managers in a telehealth setting [81]. As telehealth medical services have garnered much attention, nurse practitioners have played an increasingly important role in supporting various online care services in which multiple technologies need to be operated appropriately. Our results demonstrate that care providers’ adaptive use of technology can help predict telehealth care performance, and therefore, more consideration should be given to the role of intermediary care providers in the care process between patients and specialists. As organizational structures influence both offline and online meeting outcomes [99], health practitioners need to focus on organization-level strategies to enhance care providers’ online facilitation by examining the gaps that they have experienced using a variety of technologies across multiple life domains and providing relevant education in the use of focal technology [31,100]. Moreover, this study can be beneficial to telehealth designers and developers in terms of the design of HITs. Prior studies have documented that telehealth apps’ design of features, icons, and terminologies is important and that care providers expect all of these to function as intended [14]. As health app developers continue to add new features to stay abreast of rapidly changing health care trends, it is important for them to consider health care consumers’ needs and users’ familiarity and comfort with the existing features that are evolving across a wide range of technologies and systems [101].

### Conclusions

This study investigated frontline care providers’ unique role in e-consultation with specialists. By regarding the care providers explicitly as virtual meeting facilitators, we tested the association between their adaptive use of multiple telehealth-related technologies and virtual service performance. Care providers’ standard use of telehealth technologies was shown to be a salient factor that predicts success in virtual service, while the innovative use of telehealth technologies remained insignificant. Among their personal characteristics, the habits and computer self-efficacy that care providers acquired and developed in nonwork settings stimulated and enhanced their willingness to use multiple telehealth technologies in standard and creative ways.

## Appendix

Multimedia Appendix 1 Survey scenario.

Multimedia Appendix 2 Survey instruments.

## Abbreviations

AST: adaptive structuration theory
AVE: average variance extracted
E-consultation: electronic consultation
HCI: human computer interaction
HIT: health information technology
ICT: information and communication technology
IS: information systems
IT: information technology
PLS: partial least squares
SEM: structural equation modeling
