# Title Correction: The Association Between Willingness of Frontline Care Providers’ to Adaptively Use Telehealth Technology and Virtual Service Performance in Provider-to-Provider Communication: Quantitative Study

**DOI:** 10.2196/17123

**Published:** 2019-11-19

**Authors:** Hyeyoung Hah, Deana Goldin, Sejin Ha

**Affiliations:** 1 Department of Information Systems and Business Analytics Florida International University Miami, FL United States; 2 Nicole Wertheim College of Nursing & Health Sciences Florida International University Miami, FL United States; 3 Retail, Hospitality, and Tourism Management College of Education, Health, and Human Sciences The University of Tennessee, Knoxville Knoxville, TN United States

The title of this paper (J Med Internet Res 2019;21(8):e15087) has been changed from “The Association Between Willingness of Frontline Care Providers’ to Adaptively Use of Telehealth Technology and Virtual Service Performance in Provider-to-Provider Communication: Quantitative Study” to “The Association Between Willingness of Frontline Care Providers’ to Adaptively Use Telehealth Technology and Virtual Service Performance in Provider-to-Provider Communication: Quantitative Study”.

The corrections will appear in the online version of the paper on the JMIR website on November 19, 2019, together with the publication of this correction notice. Because this was made after submission to PubMed, PubMed Central, and other full-text repositories, the corrected article also has been resubmitted to those repositories.

