# Supplementary material for: The Association Between Willingness of Frontline Care Providers’ to Adaptively Use Telehealth Technology and Virtual Service Performance in Provider-to-Provider Communication: Quantitative Study
Source: J Med Internet Res. 2019 Aug 29;21(8):e15087. doi: 10.2196/15087 (PMC6740163; doi:10.2196/15087)
Supplement: Multimedia Appendix 1 [file jmir_v21i8e15087_app1.pdf]

Multimedia Appendix 2

Textbox 2. Survey Scenario

Assume that you are part of a telehealth team to use new telehealth app on mobile devices. Your role is to manage patients follow them up and collaborate with other healthcare team members. For example, you, the Nurse Practitioner, have a scheduled online visit with a patient who makes an online appointment to take place via video conferencing. The patient plans to use a smart phone. As the Nurse Practitioner, after reviewing the patient's medical record, all digital images in the chart, and continue to monitor the patient remotely for a time period, you decide to consult with a specialist via a telehealth technology of your choice to discuss the management of the patient's health condition.
