# Supplementary material for: The Association Between Willingness of Frontline Care Providers’ to Adaptively Use Telehealth Technology and Virtual Service Performance in Provider-to-Provider Communication: Quantitative Study
Source: J Med Internet Res. 2019 Aug 29;21(8):e15087. doi: 10.2196/15087 (PMC6740163; doi:10.2196/15087)
Supplement: Multimedia Appendix 2 [file jmir_v21i8e15087_app2.pdf]

Multimedia Appendix 1

Textbox 1. Research instruments

Adaptive technology use (adapted from Schmitz, Teng, and Webb 2016)
Exploratory technology adaptation
1. I am willing to experiment with new features on telehealth technology
2.I am willing to change the settings/preferences on telehealth technology to alter the way I interact with it
3.I am willing to take advantage of the adaptability of the features available on telehealth technology as they were intended to be used.
4.I am willing to exploit features of telehealth technology for standard usage

Exploratory technology adaptation (adapted from Schmitz, Teng, and Webb 2016)
1.I am willing to develop a way of using telehealth technology which deviates from the standard usage
2.I am willing to use at least one telehealth technology feature or capability in an unusual manner which the vendor does not encourage.
3.I am willing to modify some setting or configuration in telehealth technology to use it in a nonstandard way.
4.I am willing to explore adapting features of telehealth technology for nonstandard usage.

Virtual service performance (adapted from Goodhue and Thompson 1995)
1.I believe that telehealth technology can increase my overall performance for care tasks.
2.I believe that telehealth technology can increase my effectiveness with care tasks.
3.With telehealth technology, I believe I can work more efficiently for managing care tasks.
4.I believe that telehealth technology can increase the quality of dealing with care tasks.
5.I believe that telehealth technology systems can decrease error rates in communication and information sharing with others.

Personal innovativeness with IT (adopted from Agarwal and Prasad 1998)
1.If I heard about a new information technology, I would look for ways to experiment with it.
2.In general, I am hesitant to try out new information technologies.
3.Among my peers, I am usually the first to try out new information technologies.
4.I like to experiment with new information technologies.

Computer Self Efficacy (adopted from Compeau and Higgins 1995)
I could complete the health care task using health information technology . . .

1. . . . if there was no one around to tell me what to do as I go
2. . . . if I had just the built-in help menu for assistance.
3. . . . if someone showed me how to do it first.
4. . . . if I had used similar apps before this one to do the same job.

Habit (adopted from Venkatesh, Morris, Davis, and Davis 2003)

1.The use of mobile apps has become a habit for me

2.I am addicted to using mobile apps

3.I must use mobile apps

4.Using mobile apps has become natural to me
